# Supplementary material for: WFS1 functions in ER export of vesicular cargo proteins in pancreatic β-cells
Source: Nat Commun. 2021 Nov 30;12:6996. doi: 10.1038/s41467-021-27344-y (PMC8632972; doi:10.1038/s41467-021-27344-y)
Supplement: Supplementary file 1 — Supplementary information. [file 41467_2021_27344_MOESM1_ESM.pdf]

Supplementary Information

WFS1 functions in ER export of vesicular cargo proteins in pancreatic β-cells

Linlin Wang[1], Hongyang Liu[2], Xiaofei Zhang[3, 4], Eli Song[2, 3], You Wang[2], Tao Xu[1, 2, 3, 5]*, Zonghong Li[1]*

Supplementary information includes the following:

Supplementary Figures 1-7

Supplementary Tables 1-3

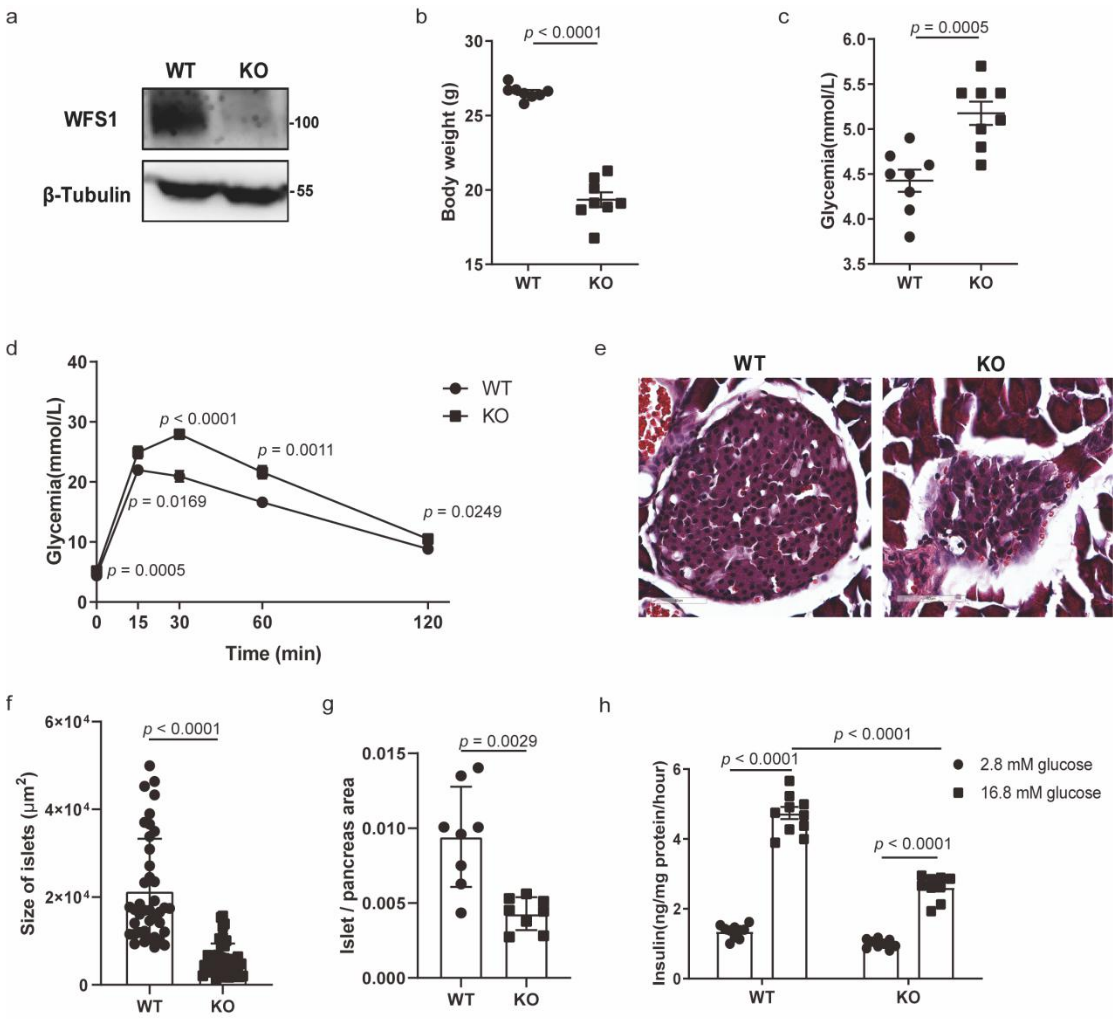

Supplementary Fig. 1: The phenotype of WFS1-deficient mice. a, IB analysis of WFS1 protein in islets derived from the WT and *Wfs1* KO mice; β-tubulin was used as the loading control. n = 3 independent experiments. b, Comparison of body weight of the WT and *Wfs1* KO mice at 8 weeks. WT/KO = 8/8, male. c, Comparison of fasted blood glucose of the WT and *Wfs1* KO mice. WT/KO = 8/8, male. d, Blood glucose during the GTT in the WT and *Wfs1* KO mice. I.p. administration of 2 g/kg body weight glucose. WT/KO = 8/8. e, Representative images of islets stained with haematoxylin and eosin in pancreatic sections of the WT and *Wfs1* KO mice. Scale bar: 60 μm. f, Comparison of islet size between the WT and *Wfs1* KO mice. WT/KO = 8/8, 5 slices per mouse. g, Comparison of islet size per pancreas between the WT and *Wfs1* KO mice. WT/KO = 8/8. **h**, Glucose-stimulated insulin secretion *in vitro* islets isolated from the WT and *Wfs1* KO mice. WT/KO = 10/9 independent experiments. All the data are presented as the mean ± s.e.m. $p < 0.05$, significant, using a two-tailed Student's t-test. Source data are provided as a Source Data file.

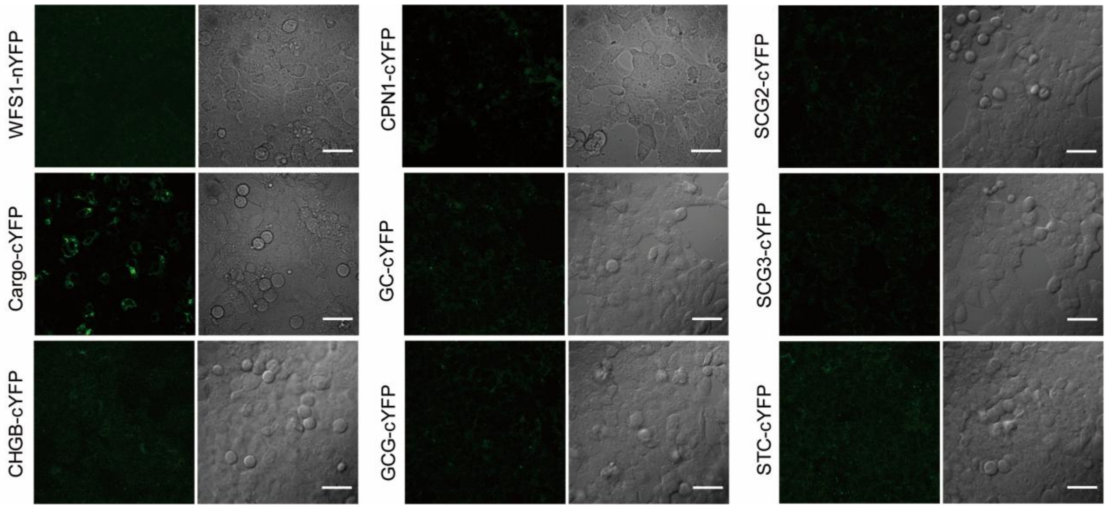

Supplementary Fig. 2: Representative live images of reconstituted BiFC fluorescence between WFS1-tagged nYFP and vesicular cargo protein-tagged cYFP. HEK-293T cells pairwise expressing WFS1-tagged nYFP and CHGB, CPN1, GC, GCG, SCG2, SCG3 or STC-tagged cYFP were exposed to low-temperature treatment to allow for fluorophore maturation. The green fluorescence shows reconstitution of YFP as an indicator of protein–protein interactions. Scale bar, 40 μm. n = 3 independent experiments.

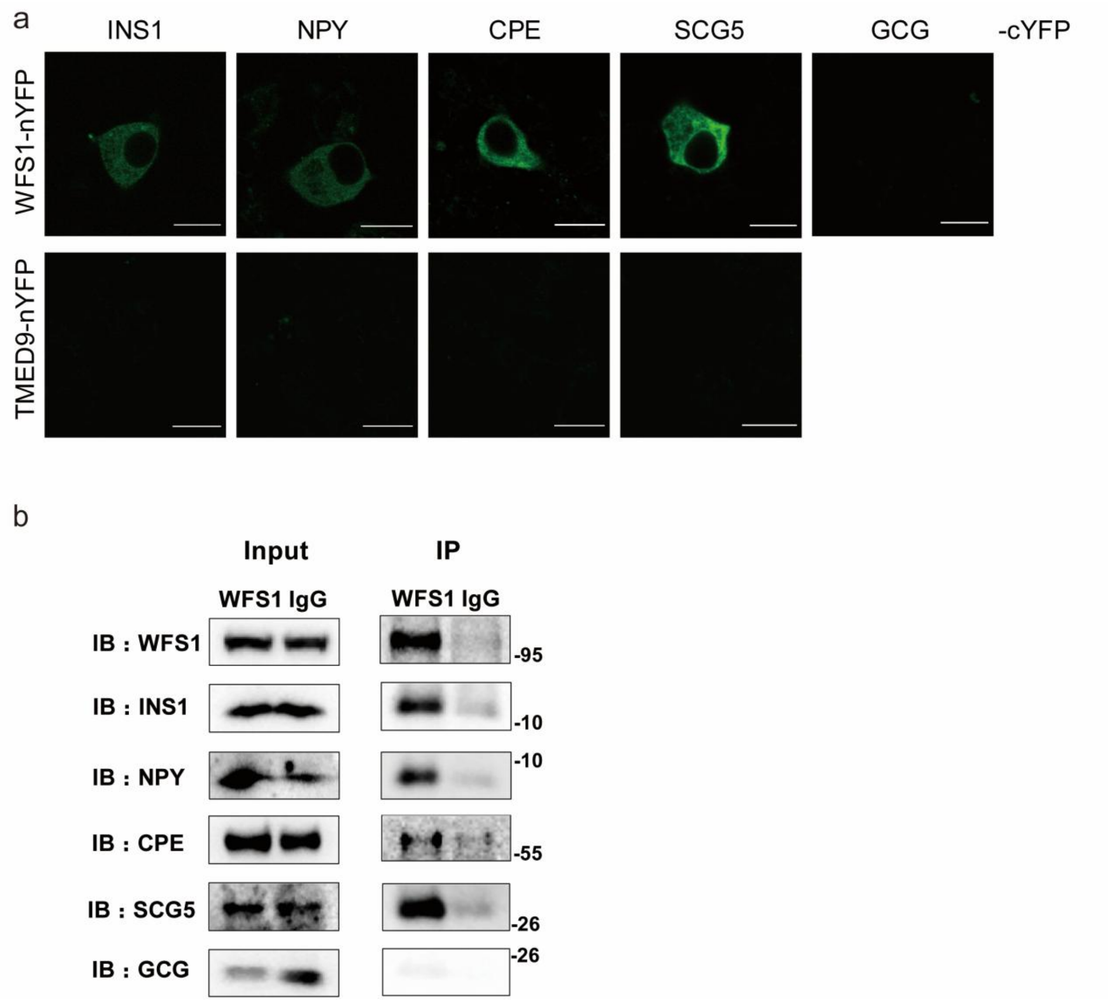

Supplementary Fig. 3: WFS1 interacts with vesicular cargo proteins in INS1 cells. a, Representative live imaging of reconstituted BiFC fluorescence between WFS1-tagged nYFP or nYFP-tagged TMED9, and vesicular cargo protein (INS1, NPY, CPE, SCG5 and negative control GCG)-tagged cYFP. Green fluorescence shows reconstitution of YFP as an indicator of protein–protein interactions. n = 3 independent experiments. Scale bar, 10 μm. b, IP analysis of the interaction of WFS1 with endogenous vesicular cargo proteins in INS1 cells. The immunoprecipitates pulled down by the WFS1 antibody were analysed by IB with the indicated antibodies. GCG was used as a negative control. Input represents 5% of the total cell extract used for immunoprecipitation. Molecular weights are in kDa. n = 3 independent experiments. Source data are provided as a Source Data file.

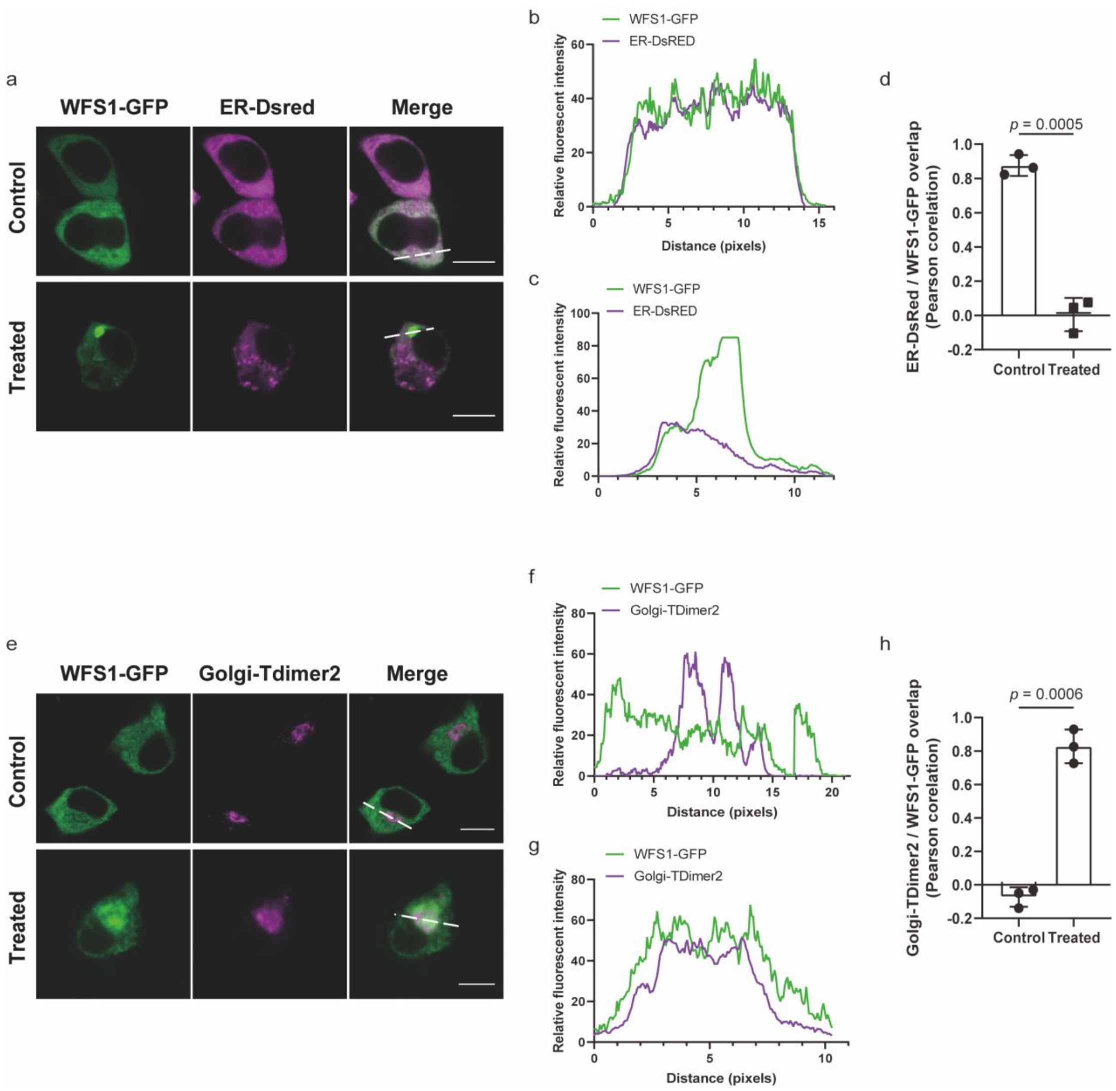

Supplementary Fig. 4: WFS1 traffics from the ER to the Golgi in INS1 cells. **a**-d, Confocal microscopy analysis of colocalization of WFS1-GFP and ER-Dsred in INS1 cells treated with normal (37 °C) or low temperature (30 °C) (**a**). Trace outline is used for line-scan (white dashed line) analysis of the relative fluorescence intensity of WFS1 and ER signals after treatment with normal (37 °C, b) or low temperature (30 °C, c). Signal overlap was quantified by Pearson correlation analysis of n = 3 independent experiments (d). Scale bar, 5 μm. **e**-h, Confocal microscopy analysis of colocalization of WFS1-GFP and Golgi-TDimer2 in HEK-293T cells treated with normal (37 °C) or low temperature (30 °C) (e). Trace outline is used for line-scan (white dashed line) analysis of the relative fluorescence intensity of WFS1 and Golgi signals after treatment with normal (37 °C, **f**) or low temperature (30 °C, **g**). Signal overlap was quantified by Pearson correlation analysis of n = 3 independent experiments (h). Scale bar, 5 μm. All the data are presented as the mean ± s.e.m. $p < 0.05$, significant, using a two-tailed Student's t-test. Source data are provided as a Source Data file.

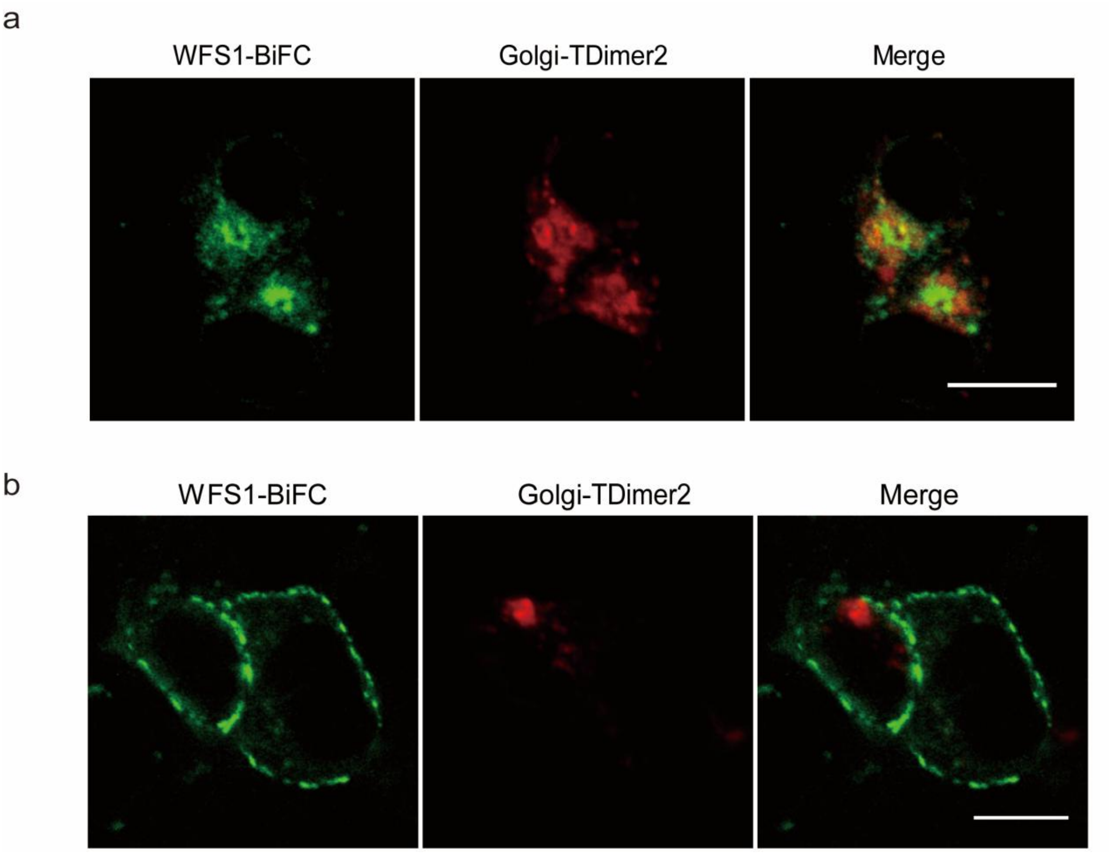

Supplementary Fig. 5: Trafficking of homodimer WFS1 in the secretory pathway. a, Confocal microscopy showing the colocalization of homodimer WFS1 (BiFC) and Golgi-TDimer2 (Golgi marker) in HEK-293T cells exposed to low-temperature treatment. n = 3 independent experiments. Scale bar, 10 μm. b, Confocal microscopy showing homodimer WFS1 trafficking to the plasma membrane in HEK-293T cells after recovery to normal temperature (37 °C) for 2 hrs. n = 3 independent experiments. Scale bar, 10 μm.

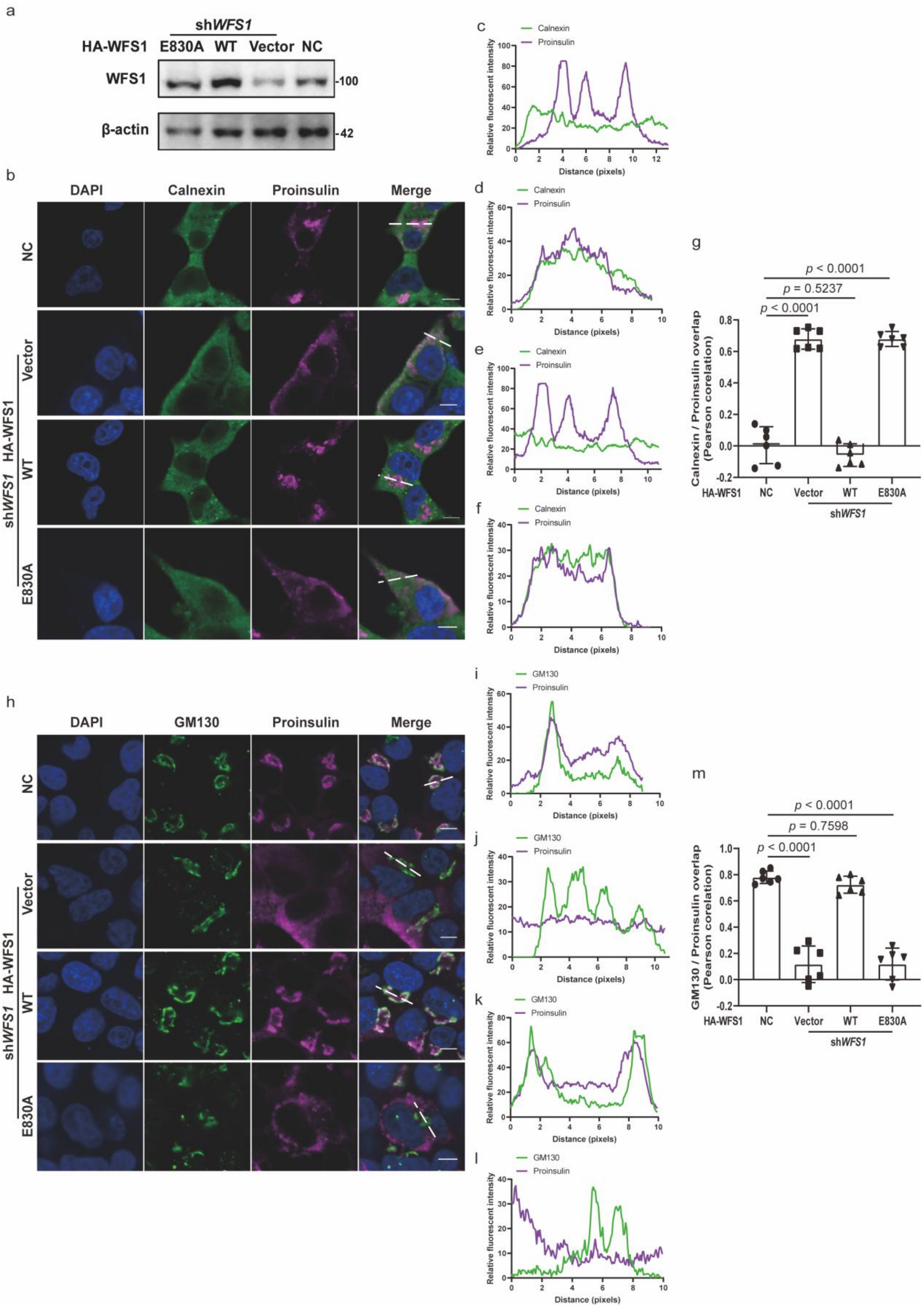

Supplementary Fig. 6: The effect of E830A mutant WFS1 on proinsulin distribution. a, IB analysis of WFS1 protein in scrambled (NC), sh*Wfs1*, rescued WT-*Wfs1* and E830A mutant *Wfs1* INS1 cells; β-actin was used as the loading control. n = 3 independent experiments. b-m, Confocal microscopy analysis of colocalization of proinsulin with calnexin (b-g) or GM130 (h-m). Trace outline is used for line-scan (white dashed line) analysis of the relative fluorescence intensities of proinsulin with calnexin or GM130

signals. Signal overlap was quantified by Pearson correlation analysis. n = 3 independent experiments, n = 6 independent images quantified. Scale bar, 5 μm. All the data are presented as the mean ± s.e.m. $p < 0.05$, significant, using a two-tailed Student's t-test. Source data are provided as a Source Data file.

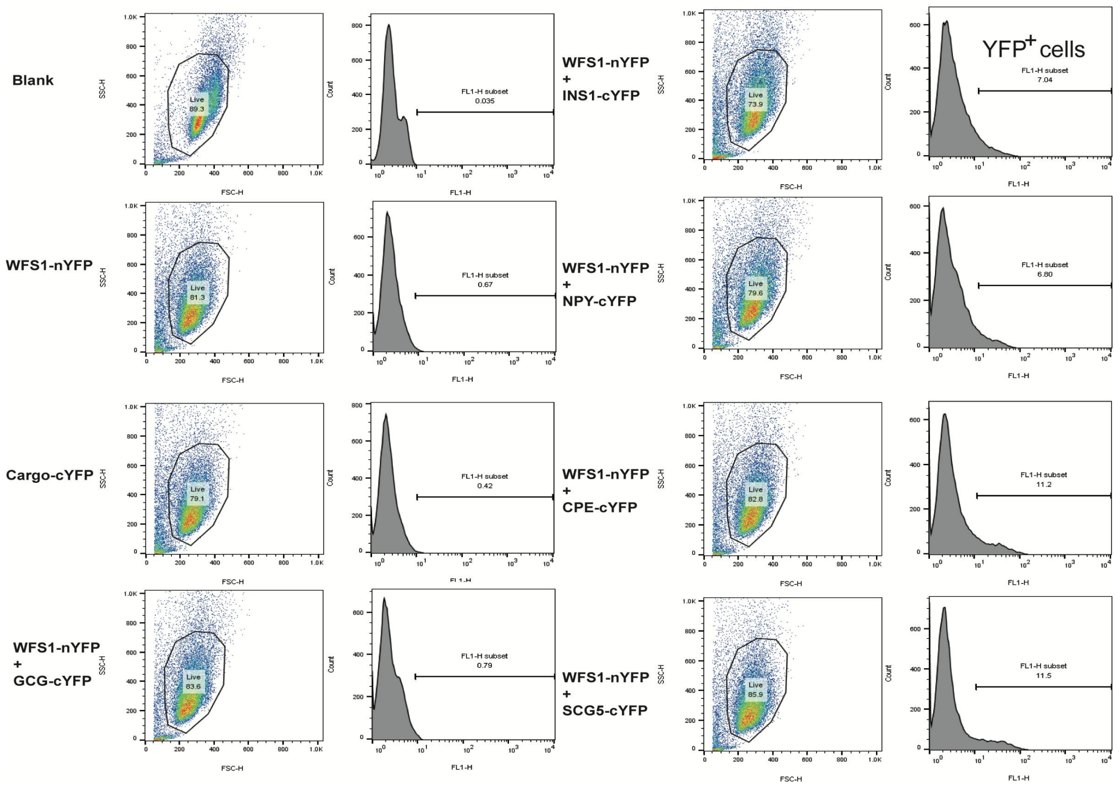

Supplementary Fig. 7: Gating strategies used for flow cytometry. Cells were selected in the FSC-H/SSC-H dot plot to remove debris. Untransfected HEK-293T cells, WFS1-nYFP transfected HEK-293T cells or cargo-cYFP transfected HEK-293T cells as the negative control sample to gate the YFP^- and YFP^+ cells.

Supplementary Table 1. The oligonucleotides are used for *Wfs1* KO mice.

Primer name	Sequence
sgRNA1	CAGGTCCCATGCATCCCCCA
sgRNA2	CTGTAAAGTGCTCTTCGTGA
WFS1-KO-tF1	GGCTGAAGTAAGCTCTATTCAGGAC
WFS1-KO-tR1	GAGCCCAGTTTGGACATGCTATAC
WFS1-WT-tF1	TTGGACAGTGTAGTCCGTCTCAG
WFS1-WT-tR1	AATCACCTGTGCCTGCTTGAG
WFS1-S1-tF1	ACCCTCAACAAAGGCTTGGGAAC
WFS1-S1-tR1	CTCTGGAAAAGCAAGCAGGGTC
WFS1-S1-tF2	TCTGAGCCAATCCCCTATGTGC
WFS1-S1-tR2	CATGAAGACGCTATTGGCCTGG
WFS1-S1-tF3	AAATCAGCACCAGTGGGATGAC
WFS1-S1-tR3	CTGAGGCAGATAAATGAGGGAGTG
WFS1-S2-tF1	CCTATGTACGAACAGACAGGTATCCC
WFS1-S2-tR1	CACTCACCCACTCTGACTTCCCTAC
WFS1-S2-tF2	ACCCTATCCTTCCTTCCCATAGTG
WFS1-S2-tR2	ATGCCTGCTGGGATTTGGTAC
WFS1-S2-tF3	GGGAGAGTGCTGTGTTTAGAGGCA
WFS1-S2-tR3	GAACCCTGTTTTGAGTCTATGTGTCC

Supplementary Table 2. The oligonucleotides are used for cloning.

Primer name	Sequence
WFS1-F	GCGTTTAAACTTAAGCTTATGAGCTCAGGCACCCCAC
WFS1-nYFP-R	GCAATGGAACGGGATCC GGCGGCAGACAGGAATGGG
WFS1-cYFP-R	GCAGGCGGGCCTGGATCCGGCGGCAGACAGGAATGGG
WFS1-ΔCT-R	GCAATGGAACGGGATCCCCGGTACACGTAGAACCAGC
WFS1-ΔNT-F	GCGTTTAAACTTAAGCTTATGATCAAAGAGTACCTGATTGACG
WFS1-ΔNT-R	GCAATGGAACGGGATCC GGCGGCAGACAGGAATGGG
HA-WFS1-F	GCGTTTAAACTTAAGCTTTACCCATACGACGTCCCAGACTACGC TATGAGCTCAGGCACCCCA
HA-WFS1-R	CACACTGGACTAGTGGATCC TCAGGCGGCAGACAGGAA
HA-ΔNT-WFS1-F	GCGTTTAAACTTAAGCTTTACCCATACGACGTCCCAGACTACGC TATGATCAAAGAGTACCTGATTG
HA-ΔNT-WFS1-R	CACACTGGACTAGTGGATCC TCAGGCGGCAGACAGGAA
HA-ΔCT-WFS1-F	GCGTTTAAACTTAAGCTTTACCCATACGACGTCCCAGACTACGC TATGAGCTCAGGCACCCCA
HA-ΔCT-WFS1-R	CACACTGGACTAGTGGATCCTCACCGGTACACGTAGAACCAGC
G695V-F	CTGTGCAGCCACCTGGAGGTCCACA
G695V-R	CTGTCCACGTGACCCTGTGGACCTCCAGGTGGCT
P724L-F	GTCGGCCATCAACATGCTCCTGTTCTTCCTGG
P724L-R	ATCCAGTCACCCAGGAAGAACAGGAGCATGT
E809K-F	CTGCGAGCCAGCAGC AAG TTCAAGGACGTGCTG
E809K-R	CAGCACGTCCTTGAACTTGCTGCTGGCTCGCAG
E830A-F	CATAGAGTTCAGCACCATCCTCGCGGGCCGCCTGGGTAGC
E830A-R	GCTACCCAGGCGGCCCGCGAGGATGGTGCTGAACTCTATG
E158K-F	ACCGGAAAGGCATCACTTCTAAGAATGAGGCCGAGGTGAAGCA
E158K-R	TGCTTCACCTCGGCCTCATTCTTAGAAGTGATGCCTTTCCGGT
E169K-F	AGGTGAAGCAGCTATCCTCTAAGACCGACCTGGAAAGGGCAGT
E169K-R	ACTGCCCTTTCCAGGTCGGTCTTAGAGGATAGCTGCTTCACCT
INS1-cYFP-F	GCGTTTAAACTTAAGCTT ATGGCCCTGTGGATGCGC
INS1-cYFP-R	GCAGGCGGGCCTGGATCCGTTGCAGTAGTTCTCCAGTT
GCG-cYFP-F	GCGTTTAAACTTAAGCTT ATGAAGACCGTTTACATCGT
GCG-cYFP-R	GCAGGCGGGCCTGGATCCTTTCTTGTCAGTGATCTTGGT
NPY-cYFP-F	GCGTTTAAACTTAAGCTT ATGATGCTAGGTAACAAACG
NPY-cYFP-R	GCAGGCGGGCCTGGATCCCCACATGGAAGGGTCTTC
CPE-cYFP-F	GCGTTTAAACTTAAGCTT ATGGCCGGGCGCGGAGGA
CPE-cYFP-R	GCAGGCGGGCCTGGATCCAAAATTCAAAGTCTCTGACATCATT
SCG2-cYFP-F	GCGTTTAAACTTAAGCTT ATGACTGAATCGAAGGCTTAC
SCG2-cYFP-R	GCAGGCGGGCCTGGATCCCATGTTTTCCATGGCCCGT
SCG3-cYFP-F	GCGTTTAAACTTAAGCTT ATGGGGTTCCTTTGGACAGG
SCG3-cYFP-R	GCAGGCGGGCCTGGATCCCAGGCTGCTGTAGATGCGT
SCG5-cYFP-F	GCGTTTAAACTTAAGCTT ATGACCTCAAGGATGGCCAT

SCG5-cYFP-R	GCAGGCGGGCCTGGATCCTTCTGGCTCCTTCTCCTCTT
CHGB-cYFP-F	GCGTTTAAACTTAAGCTT ATGCAGCGGGCCATGCTCCT
CHGB-cYFP-R	GCAGGCGGGCCTGGATCCGCCCCGCTGGCTGAACTTTTC
STC-cYFP-F	GCGTTTAAACTTAAGCTT ATGCTCCAAAACTCAGCAGT
STC-cYFP-R	GCAGGCGGGCCTGGATCCCGCATTCTCTTGGGAGGTG
GC-cYFP-F	GCGTTTAAACTTAAGCTT ATGAAGAGGGTTCTGGTTCT
GC-cYFP-R	GCAGGCGGGCCTGGATCCGGACTGCAGGATGTCTCTC
CPN1-cYFP-F	GCGTTTAAACTTAAGCTT ATGCCAGACCTGCCCTCAG
CPN1-cYFP-R	GCAGGCGGGCCTGGATCCTGCAGGGCCCCTGTGCC
nYFP-TMED9	GCGTTTAAACTTAAGCTT ATGGCTGCGGTGCGAGG
nYFP-TMED9	GCAATGGAACGGGATCCCACCAACTTCTTGGCTTCAAAA
sumo-SEC24A-F	GAACAGATTGGTGGATCCATGGCCCAGCCCAGGATC
sumo-SEC24A-R	CAGTGGTGGTGCTCGAGTCACTTGTTCACTTGCT
sumo-SEC24B-F	GAACAGATTGGTGGATCC ATGTCGGCCCCCGCCGGGT
sumo-SEC24B-R	TCAGTGGTGGTGCTCGAG TCACTTACAAACCTGCTG
sumo-SEC24C-F	GAACAGATTGGTGGATCC ATGAATGTCAACCAGTCA
sumo-SEC24C-R	TCAGTGGTGGTGCTCGAG TTAGCTCAGCAGCTGCCG
sumo-SEC23A-F	GAACAGATTGGTGGATCC ATGACAACCTATTTGGAA
sumo-SEC23A-R	TCAGTGGTGGTGCTCGAG TCAAGCAGCACTTGACAC
sumo-SEC23B-F	GAACAGATTGGTGGATCC ATGGCAACATATCTGGAA
sumo-SEC23B-R	TCAGTGGTGGTGCTCGAG TTAAGAGGCACTGGACAC
SEC24A-Myc-F	GCTAGCGTTTAAACTTAAGCTTATGGCCCAGCCCAGGATCCCG
SEC24A-Myc-R	CACACTGGACTAGTGGATCCTCACAGATCCTCTTCTGAGATGAG TTTTTGTTCCTCGAGCTTGTTCACTTGCTG
SEC24B-Myc-F	GCTAGCGTTTAAACTTAAGCTTATGTCGGCCCCCGCCGGGTCC
SEC24B-Myc-R	CACACTGGACTAGTGGATCCTCACAGATCCTCTTCTGAGATGAG TTTTTGTTCCTCGAG CTTACAAACCTGCTG
SEC24C-Myc-F	GCTAGCGTTTAAACTTAAGCTTATGAATGTCAACCAGTCAGC
SEC24C-Myc-R	CACACTGGACTAGTGGATCCTCACAGATCCTCTTCTGAGATGAG TTTTTGTTCCTCGAG GCTCAGCAGCTGCCG
SEC23A-Myc-F	GCTAGCGTTTAAACTTAAGCTTATGACAACCTATTTGGAATTT
SEC23A-Myc-R	CACACTGGACTAGTGGATCCTCACAGATCCTCTTCTGAGATGAG TTTTTGTTCCTCGAG AGCAGCACTTGACAC
SEC23B-Myc-F	GCTAGCGTTTAAACTTAAGCTTATGGCAACATATCTGGAATTC
SEC23B-Myc-R	CACACTGGACTAGTGGATCCTCACAGATCCTCTTCTGAGATGAG TTTTTGTTCCTCGAG AGAGGCACTGGACAC
Scrambled	GTTCTCCGAACGTGTCACGT
WFS1-shRNA-1	GGAGCAGGACAAGATTGAACC
WFS1-shRNA-2	GCCGAGAAGGGACAGATAAAG
WFS1-shRNA-3	GCAGTGAATCCAAGAACTACA
WFS1-shRNA-4	GCAACCTAACCATCGACTTCT

Supplementary Table 3. The antibodies are used in this study.

Antibodies	Company name, catalog number, dilution
WFS1 for IB	Proteintech, 26995-1-AP, 1:1000
WFS1 for IP	LSBio, LS-C661616, 1:200
β-Tubulin	Sungene biotech, KM9003T, 1:2000
Proinsulin	Hytest, CCI-17, 1:200 for IF or 1:2000 for IB
Insulin	Abcam, ab7842,1:200 for IF or 1:1000 for IB
Calnexin	Biorbyt, orb46780,1:200 for IF or 1:1000 for IB
GM130	Invitrogen, PA5-95727, 1:200 for IF or 1:1000 for IB
HA-tag for IB	Cell Signaling Technology, 2367S, 1:2000
HA-tag for IP (Beads)	Thermo Scientific, 88836, 100 μL
GFP-tag	Proteintech, 50430-2-AP, 1:1000
Myc-tag	Cell Signaling Technology, 2278S, 1:200 for IF
His-tag	Proteintech, 66005-1-Ig, 1:1000
β-Actin	Proteintech, 66009-1-Ig, 1:2000
NPY	Cell Signaling Technology, 11976S, 1:1000
CPE	BD Biosciences, 610758, 1:1000
SCG5	Invitrogen, PA5-68244, 1:300
GCG	Abcam, ab92517, 1:1000
Goat anti-mouse IgG (H+L), HRP conjugate	Proteintech, SA00001-1, 1:5000
Goat Anti-Rabbit IgG(H+L), HRP conjugate	Proteintech, SA00001-2, 1:5000
Goat anti-Rabbit IgG (H+L) Cross-Adsorbed Secondary Antibody, Alexa Fluor 488	Invitrogen, A-11008, 1:500
Goat anti-Mouse IgG (H+L) Highly Cross-Adsorbed Secondary Antibody, Alexa Fluor Plus 647	Invitrogen, A32728, 1:500
Rhodamine (TRITC) AffiniPure Goat Anti-Guinea Pig IgG (H+L),	Jackson IR, 106-025-003, 1:500
