# Supplementary material for: WFS1 functions in ER export of vesicular cargo proteins in pancreatic β-cells
Source: Nat Commun. 2021 Nov 30;12:6996. doi: 10.1038/s41467-021-27344-y (PMC8632972; doi:10.1038/s41467-021-27344-y)
Supplement: Supplementary file 3 — Reporting summary. [file 41467_2021_27344_MOESM3_ESM.pdf]

nature research

Corresponding author(s): Zonghong Li

Last updated by author(s): Oct 30, 2021

Reporting Summary

Nature Research wishes to improve the reproducibility of the work that we publish. This form provides structure for consistency and transparency in reporting. For further information on Nature Research policies, see our Editorial Policies and the Editorial Policy Checklist.

Statistics

For all statistical analyses, confirm that the following items are present in the figure legend, table legend, main text, or Methods section.

n/a	Confirmed	
☐	☒	The exact sample size (n) for each experimental group/condition, given as a discrete number and unit of measurement
☐	☒	A statement on whether measurements were taken from distinct samples or whether the same sample was measured repeatedly
☐	☒	The statistical test(s) used AND whether they are one- or two-sided<br>*Only common tests should be described solely by name; describe more complex techniques in the Methods section.*
☒	☐	A description of all covariates tested
☒	☐	A description of any assumptions or corrections, such as tests of normality and adjustment for multiple comparisons
☐	☒	A full description of the statistical parameters including central tendency (e.g. means) or other basic estimates (e.g. regression coefficient) AND variation (e.g. standard deviation) or associated estimates of uncertainty (e.g. confidence intervals)
☐	☒	For null hypothesis testing, the test statistic (e.g. F, t, r) with confidence intervals, effect sizes, degrees of freedom and P value noted<br>*Give P values as exact values whenever suitable.*
☒	☐	For Bayesian analysis, information on the choice of priors and Markov chain Monte Carlo settings
☒	☐	For hierarchical and complex designs, identification of the appropriate level for tests and full reporting of outcomes
☐	☒	Estimates of effect sizes (e.g. Cohen's d, Pearson's r), indicating how they were calculated

Our web collection on statistics for biologists contains articles on many of the points above.

Software and code

Policy information about availability of computer code

Data collection	FACS data were acquired on BD FACSCalibur with BD CellQuest Pro™ Software (Version 6.0). Confocal image data were acquired on Olympus FV12000 with FV10-ASW software (Version 02.01.02.05). CRISPOR (Version 4.99)
Data analysis	GraphPad Prism (Version 8.0.2), FlowJo (Version 10.5.3 win64), CRISPOR (Version 4.99), ImageJ Fiji ((Version 1.8.0)

For manuscripts utilizing custom algorithms or software that are central to the research but not yet described in published literature, software must be made available to editors and reviewers. We strongly encourage code deposition in a community repository (e.g. GitHub). See the Nature Research guidelines for submitting code & software for further information.

Data

Policy information about availability of data

All manuscripts must include a data availability statement. This statement should provide the following information, where applicable:

- Accession codes, unique identifiers, or web links for publicly available datasets
- A list of figures that have associated raw data
- A description of any restrictions on data availability

The authors declare that all the data supporting the findings of this study and the source data are available within the article.

Field-specific reporting

Please select the one below that is the best fit for your research. If you are not sure, read the appropriate sections before making your selection.

☒ Life sciences ☐ Behavioural & social sciences ☐ Ecological, evolutionary & environmental sciences

For a reference copy of the document with all sections, see nature.com/documents/nr-reporting-summary-flat.pdf

Life sciences study design

All studies must disclose on these points even when the disclosure is negative.

Sample size	Sample size for in vivo and in vitro experiments is chose based on previous studies in order to ensure adequate statistics power ($n \geq 3$). All findings are reliably reproduced. The sample sizes and number of repeats are defined in each figure legends.
Data exclusions	No data were excluded from the analysis
Replication	All experimental findings were reliably obtained in multiple biological and technical replicates. Results were verified with minimum of three independent experiments.
Randomization	Randomization with blinding was used for mice experiments.
Blinding	The investigators were blinded to group allocation during data collection and analysis.

Reporting for specific materials, systems and methods

We require information from authors about some types of materials, experimental systems and methods used in many studies. Here, indicate whether each material, system or method listed is relevant to your study. If you are not sure if a list item applies to your research, read the appropriate section before selecting a response.

Materials & experimental systems

n/a	Involved in the study	
☐	☒	Antibodies
☐	☒	Eukaryotic cell lines
☒	☐	Palaeontology and archaeology
☐	☒	Animals and other organisms
☒	☐	Human research participants
☒	☐	Clinical data
☒	☐	Dual use research of concern

Methods

n/a	Involved in the study	
☒	☐	ChIP-seq
☐	☒	Flow cytometry
☒	☐	MRI-based neuroimaging

Antibodies

Antibodies used

WFS1 for IB Proteintech, 26995-1-AP, 1:1000
WFS1 for IP LSBio, LS-C661616, 1:200
β-Tubulin Sungene biotech, KM9003T, 1:2000
Proinsulin Hytest, CCI-17, 1:200 for IF or 1:2000 for IB
Insulin Abcam, ab7842,1:200 for IF or 1:1000 for IB
Calnexin Biorbyt, orb46780,1:200 for IF or 1:1000 for IB
GM130 Abcam, ab52649, 1:200 for IF or 1:1000 for IB
HA-tag Cell Signaling Technology, 2367S, 1:200 for IP or 1:2000 for IB
GFP-tag Proteintech, 50430-2-AP, 1:1000
Myc-tag Cell Signaling Technology, 2278S, 1:200 for IF
His-tag Proteintech, 66005-1-Ig, 1:1000
β-Actin Proteintech, 66009-1-Ig, 1:2000
NPY Cell Signaling Technology, 11976S, 1:1000
CPE BD Biosciences, 610758, 1:1000
SCG5 Invitrogen, PA5-68244, 1:300
GCG Abcam, ab92517, 1:1000
Goat anti-mouse IgG (H+L), HRP conjugate Proteintech, SA00001-1, 1:5000
Goat Anti-Rabbit IgG(H+L), HRP conjugate Proteintech, SA00001-2, 1:5000
Goat anti-Rabbit IgG (H+L) Cross-Adsorbed Secondary Antibody, Alexa Fluor 488 Invitrogen, A-11008, 1:500
Goat anti-Mouse IgG (H+L) Highly Cross-Adsorbed Secondary Antibody, Alexa Fluor Plus 647 Invitrogen, A32728, 1:500
Rhodamine (TRITC) AffiniPure Goat Anti-Guinea Pig IgG (H+L), Jackson IR, 106-025-003, 1:500

Validation	All primary antibodies used in this study were validated by the manufacture and published paper. Validation data / citations can be found on the manufacture website by searching the antibody catalog number provided in materials and methods section of our manuscript.<br><br>WFS1 for IB, applications: WB, IHC, IF, ELISA; species reactivity: Human, Mouse, Rat<br>https://www.ptglab.com/Products/WFS1-Antibody-26995-1-AP.htm<br><br>WFS1 for IP, applications: WB, IHC, IP, ELISA; species reactivity: Human<br>https://www.lsbio.com/antibodies/wfs1-antibody-elisa-ihc-ip-wb-western-ls-c661616/675584<br><br>β-Tubulin, applications: WB, IHC, IF; species reactivity: Human, Rat, Mouse, C.elegans, Fruit fly<br>http://www.sungenebiotech.com/index.php?m=Product&a=product_xq&catid=2&proid=53&prid=291&pid=724&id=1569<br><br>Proinsulin, applications: WB, IHC, ELISA; species reactivity:Rat, Mouse<br>https://shop.hytest.fi/product/rat-proinsulin-antibody<br>PDIA1/P4HB is required for efficient proinsulin maturation and ß cell health in response to diet induced obesity. Elife. 2019.<br><br>Insulin, applications: WB, IHC, IF, FC; species reactivity: Human, Rat Mouse, Syrian hamster<br>https://www.abcam.com/insulin-antibody-ab7842.html<br><br>CANX, applications: WB, IHC, IF, ELISA; species reactivity: Human<br>https://www.biorbyt.com/canx-antibody-orb46780.html<br><br>GM130, applications: WB, IF, IHC; species reactivity: Human, Mouse, Rat<br>https://www.thermofisher.com/cn/zh/antibody/product/GM130-Antibody-Polyclonal/PA5-95727<br><br>HA-Tag, applications: WB, IHC, IF, FC; species reactivity: all<br>https://www.cellsignal.com/products/primary-antibodies/ha-tag-6e2-mouse-mab/2367<br><br>HA-Tag Beads, applications: IP; species reactivity: all<br>https://www.thermofisher.cn/order/catalog/product/88836#/88836<br><br>GFP-Tag, applications: WB, IHC, IF, IP, RIP, CoIP; species reactivity: all<br>https://www.ptglab.com/products/eGFP-Antibody-50430-2-AP.htm<br><br>Myc-Tag, applications: WB, IF, IP, FC, CoIP; species reactivity: all<br>https://www.cellsignal.com/products/primary-antibodies/myc-tag-71d10-rabbit-mab/2278<br><br>His-Tag, applications: WB, IP, IHC, IF, FC, CoIP; species reactivity: all<br>https://www.ptglab.com/products/His-Tag-Antibody-66005-1-Ig.htm<br><br>Actin, applications: WB, IP, IHC, IF, FC, CoIP, ChIP; species reactivity: Human, Mouse, Rat, Hamster, Zebrafish, Monkey, Dog<br>https://www.ptglab.com/products/Pan-Actin-Antibody-66009-1-Ig.htm<br><br>Npy, applications: WB, IHC, IF; species reactivity: Human, Mouse, Rat,<br>https://www.cellsignal.com/products/primary-antibodies/neuropeptide-y-d7y5a-xp-rabbit-mab/11976<br><br>CPE, applications: WB, IHC, IF; species reactivity: Human, Mouse, Rat<br>https://www.bdbiosciences.com/en-us/products/reagents/microscopy-imaging-reagents/immunofluorescence-reagents/purified-mouse-anti-carboxypeptidase-e.610758<br><br>SCG5, applications: WB; species reactivity: Human, Mouse, Rat<br>https://www.thermofisher.cn/cn/zh/antibody/product/SCG5-Antibody-Polyclonal/PA5-68244<br><br>GCG, applications: WB, IHC; species reactivity: Human, Mouse, Rat<br>https://www.abcam.com/Glucagon-antibody-EP3070-ab92517.html

Eukaryotic cell lines

Policy information about cell lines

Cell line source(s)	HEK293T, Plat-E, and INS1 cells were obtained from the Cell Resource Center, Peking Union Medical College
Authentication	Morphological evaluation of the cells was used as authentication method. For INS1 cells, the ability of insulin secretion stimulated by glucose was used as authentication method.
Mycoplasma contamination	All the cells used in the study were not contaminated. The Mycoplasma PCR Detection Kit was used to detect the mycoplasma contamination.
Commonly misidentified lines<br>(See ICLAC register)	No commonly misidentified cell lines were used.

Animals and other organisms

Policy information about studies involving animals; ARRIVE guidelines recommended for reporting animal research

Laboratory animals	Wfs1 knockout mouse was purchased from the GemPharmatech Co., Ltd (C57BL/6JGpt background). Mice were housed in groups of three to five at 22-24 °C and 40-60% humidity, with a 12 hr light/12 hr dark cycle. Animals had access to water ad libitum. All mice used in this study were gender (male)- and age (6-8 weeks)-matched.
Wild animals	No wild animals were used in this study.
Field-collected samples	The study did not involve samples collected from the field.
Ethics oversight	All animal experiments were approved by the Animal Care Committee at the Institute of Biophysics (License No. SYXK2016-19).

Note that full information on the approval of the study protocol must also be provided in the manuscript.

Flow Cytometry

Plots

Confirm that:

☒ The axis labels state the marker and fluorochrome used (e.g. CD4-FITC).

☒ The axis scales are clearly visible. Include numbers along axes only for bottom left plot of group (a 'group' is an analysis of identical markers).

☒ All plots are contour plots with outliers or pseudocolor plots.

☒ A numerical value for number of cells or percentage (with statistics) is provided.

Methodology

Sample preparation	HEK293T cells were plated in 24-well plates and, after 24 h, transfected with 200 ng nYFP- and 200 ng cYFP-tagged constructs. After 48 h, the fluorescence of 100,000 cells per sample was determined by flow cytometry using the BD LSRFortessa™ Cell Analyzer (BD Biosciences) with the HTS autosampler device.
Instrument	BD FACSCalibur
Software	BD Cell QuestTM Pro software
Cell population abundance	No flow based sorting was performed.
Gating strategy	Cells were selected in the FSC-H/SSC-H dot plot to remove debris. Untransfected HEK-293T cells, WFS1-nYFP transfected HEK-293T cells or cargo-cYFP transfected HEK-293T cells as the negative control sample to gate the YFP- and YFP+ cells.

☒ Tick this box to confirm that a figure exemplifying the gating strategy is provided in the Supplementary Information.
